# Physical education or playtime: which is more effective at promoting physical activity in primary school children?

**DOI:** 10.1186/s13104-015-0979-1

**Published:** 2015-01-20

**Authors:** Carly Wood, Katie Hall

**Affiliations:** School of Biological Sciences, University of Essex, Wivenhoe Park, Colchester, UK

**Keywords:** Physical activity, Playtime, Physical education, Team games

## Abstract

**Background:**

School physical education (PE) and playtime provide important opportunities for physical activity (PA). However, little research has assessed PA during primary school PE using accelerometry or compared PA during different lesson types. There is also a lack of research comparing PA during PE and playtime, despite suggestions that playtime promotes more PA. The primary aim of this study was to determine which types of PE lesson are most facilitative of PA. The secondary aim was to determine whether children are more active during PE or playtime.

**Methods:**

Descriptive and fitness data were assessed in 20 children aged 8-9years from a single school. Over eight consecutive weeks PA was assessed during PE lessons, which were classified as either team games or movement activities. At the mid-week of data collection playtime PA was also assessed. PA was assessed using accelerometry and the percentage of time spent in moderate to vigorous PA (MVPA) calculated. Paired t-tests were used to compare MVPA during movement lessons and team games lessons and during PE and playtime.

**Results:**

Children spent 9.5% of PE lessons in MVPA and engaged in significantly more MVPA during team games (P < 0.001). MVPA was also significantly higher during PE than playtime (P < 0.01).

**Conclusions:**

Children do not engage in sufficient PA during PE, but are most active during team games lessons; whilst PA during playtime is lower than PE. Interventions to increase PA during both PE and playtime are therefore required. PE interventions should target games lessons as they dominate the curriculum, encourage most PA and present the greatest potential for change. Playtime interventions should encourage participation in active games through the provision of playground equipment and markings.

## Background

Physical activity (PA) during childhood is important for physical and mental health and may track into adulthood where it is protective against many chronic diseases [1-7]. However, despite the importance of PA for health, only 21% of boys and 16% of girls aged 5–15 years in the UK report meeting the recommendation of sixty minutes of daily moderate to vigorous PA (MVPA) [8].

The school environment provides children with regular opportunities to engage in PA, namely through the provision of playtime and physical education (PE) [9-12]. The aim of PE is to encourage children to take part in appropriate amounts of PA and gain the skills and knowledge to be active outside school and throughout life [13]. The diverse aims of PE present a pedagogical challenge to balancing provision of a positive experience for children whilst keeping pupils physically active and providing a ‘physical education’ [14]. Fairclough and Stratton [10] suggested that children should spend a minimum of 50% of PE lessons engaged in MVPA, however evidence suggests that children spend less than 40% of lesson time in MVPA and the majority of PE lessons in activities less intense than walking [10,15,16]. Furthermore, Sallis et al. [17] found that PE only provided 17.8 minutes of MVPA per week, contributing less than 5% to overall activity requirements. Thus, whilst PE may provide an opportunity for children to develop skills, evidence suggests that it is not achieving its aim of keeping pupils physically active.

In addition to low levels of PA during PE, evidence suggests that MVPA may significantly vary according to the type of PE lesson. As PE is concerned with providing a ‘physical education’ in addition to promoting PA [14]; it may not be surprising that some lessons are less facilitative of PA than others. Fairclough and Stratton [10] found that adolescents aged 11–14 years engaged in most MVPA during team games (e.g. football) and individual activities (e.g. athletics) and the least during individual games (e.g. badminton) and movement activities (e.g. dance). During team games pupils spent 43% of lesson time engaged in MVPA, a figure which was significantly greater than the 22% of movement activities lessons spent in MVPA [10]. A similar finding was demonstrated in children aged 5–11 years with higher levels of MVPA being performed during team games than movement activities [18]. However, neither of these studies, and indeed very few studies, have assessed MVPA during PE using accelerometry [12]. Accelerometers have been demonstrated to be a reliable and valid measure of PA in children, the output of which is highly correlated with oxygen consumption and energy expenditure [19,20]. In addition, studies have primarily focused on adolescents; very few studies have assessed PA during PE in primary school children.

In light of the low levels of PA reported during PE, attention has been turned to school playtime as means of allowing children to engage in PA [11]. In the UK, children receive up to 600 playtimes per year; thus providing a significant proportion of time where children can engage in MVPA [11,21]. However, evidence suggests that playtime currently only contributes 5-40% towards the daily activity requirement [11]. Furthermore, despite the fact that playtime might provide a more effective opportunity for PA than PE [11], comparisons to confirm this hypothesis are lacking.

The primary aim of this study was therefore to determine how active primary school children are during PE and which types of PE lesson are most facilitative of PA. The secondary aim was to determine whether children are more active during PE lessons or school playtime.

## Methods

### Participants

Participants from a local public primary school were recruited to take part in the study. The school holds 283 pupils aged 7–11 years and is classified by the Office of National Statistics [22] as in the top 30% most deprived areas in England. Twenty children from one class volunteered; including 13 boys aged 9.3 ± 0.5 years and seven girls aged 9.4 ± 0.5 years. This class and their generalist class teacher were selected by the school itself as it was felt that participation in the research would not interfere with preparations for exams or assessment. All participants provided individual assent and parental consent to take part in the study. Institutional ethical approval was granted by the University of Essex Ethics Committee.

### Procedures

Initially participants’ basic anthropometric data were collected comprising stature to the nearest 0.1 cm with the participant barefoot and mass to the nearest 0.1 kg. Body mass index and BMI Z-scores relative to the individuals’ age and sex were also calculated [23]. Participants also completed a version of the fitnessgram pacer test, which is a valid method by which to assess aerobic fitness in this age group [24].

Following the collection of anthropometric data participants’ PA was assessed during their PE lessons. Lessons were monitored over an eight week period comprising of 12 different PE lessons. The activities performed were categorised according to the characteristics of the activity, as previously defined by Fairclough and Stratton [10]. Participants engaged in team games (e.g. football) in two thirds of PE lessons and movement activities (e.g. dance) for one third of PE lessons.

For one week during the mid-point of data collection 12 participants’ also had their PA assessed during school playtime. Only 12 participants (9 males and 3 females) had their playtime PA assessed due to a limited number of accelerometers with the ability to assess MVPA using a 1-second epoch over extended periods. Playtime PA was assessed for the entire week including morning and lunch playtime, which lasted 15 minutes and 60 minutes respectively, including the time taken to eat lunch. All playtime was performed on the playground which was approximately 1700 m^2^; children were free to play on all areas of the playground, including the play structures and playground markings.

### Physical activity measurement

Participants PA was assessed using either an Actigraph GT3X or GT1M accelerometer. Accelerometers were placed on the right hip and worn during only PE and playtime. Researchers were present on each day of testing to aid the children in fitting the accelerometers. All accelerometers were set to record at a 1 second epoch and were initialised and downloaded using Actilife (version 6.9.1). The cut points of Evenson et al. [25] were applied to the data in order to determine the time spent in MVPA. Studies have identified strong agreement in the output of the two accelerometer models [26].

### Data analysis

A statistical power analysis (G Power 3.1) was conducted to determine sample size using data from Fairclough and Stratton [10] comparing MVPA during different types of PE lessons. With an alpha of 0.05 and power of 0.95, power analysis revealed that the projected sample size needed was approximately N = 14. Our sample size of N = 20 was therefore adequate to test our main hypothesis.

Data analysis was conducted using SPSS statistical analysis software (v.19). Initially Kolmogorov-Smirnov normality tests were used to ensure that data was normally distributed. Independent t-tests were used to compare anthropometric measures in boys and girls. Due to variation in the duration of PE lessons and between PE and playtime, the percentage of time spent in MVPA was calculated. A Paired samples t-test was used to compare the percent of time spent in MVPA during the two different types of PE lessons. A paired samples t-test also compared the average MVPA during PE and playtime. Statistical significance was set at P < 0.05 throughout the analysis.

## Results

There were no significant differences in anthropometric measures in boys and girls (Table 1). BMI z-scores revealed that boys had a slightly below average BMI for their age and sex and girls had a slightly above average BMI. The 20mSRT z-score revealed that boys’ fitness was slightly above average for their age and sex whilst girls’ fitness was slightly below average.

**Table 1:** **Mean ± SD descriptive anthropometric and fitness data for sample**

**Measure**	**Boys (n = 13)**	**Girls (n = 7)**	**All (n = 20)**
**Age (years)**	9.3 ± 0.5	9.4 ± 0.5	9.4 ± 0.5
**Height (m)**	1.37 ± 0.07	1.36 ± 0.07	1.37 ± 0.07
**Weight (kg)**	35.0 ± 7.0	34.8 ± 7.3	34.9 ± 6.9
**BMI (kg.m** ^**−2**^ **)**	18.5 ± 2.9	18.5 ± 2.6	18.5 ± 2.7
**BMI (Z Score)**	−0.01 ± 1.06	0.02 ± 0.97	0.00 ± 1.00
**20mSRT shuttles (no.)**	31.0 ± 10.9	30.0 ± 5.8	30.7 ± 9.4
**20mSRT speed (km.h** ^**−1**^ **)**	10.9 ± 0.7	10.2 ± 0.3	10.2 ± 0.6
**20mSRT (Z score)**	0.03 ± 1.16	−0.07 ± 0.62	0.00 ± 1.00

BMI = Body Mass Index.

**Figure 1:**
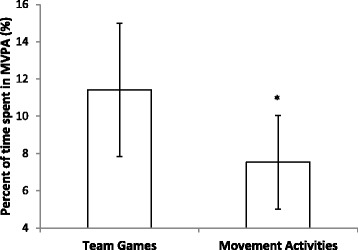
**Mean ± SD percentage of team games and movement physical education lessons spent in MVPA.** (MVPA = moderate to vigorous physical activity; *indicates a significant difference between MVPA during team games and movement lessons (P < 0.001)).

There was a significant difference between the percent of time spent in MVPA during team games and movement activity PE lessons (t(19) = −4.66; P < 0.001). MVPA was significantly higher during team games compared to movement PE lessons (Figure 1).

**Figure 2:**
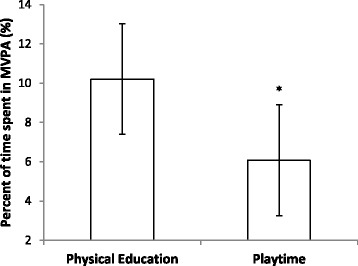
**Mean ± SD percentage of physical education and playtime spent in MVPA.** (MVPA = moderate to vigorous physical activity; *indicates a significant difference between MVPA during physical education and playtime (P < 0.001)).

There was also a significant difference between the percent of time spent in MVPA during PE and playtime (t(11) = −5.29; P < 0.001); MVPA was significantly greater during PE than playtime (Figure 2).

## Discussion

The primary aim of this study was to determine how active UK primary school children are during PE and which type of PE lesson is most facilitative of PA. To date, research has primarily focused on adolescents aged 11+ years [10,15-18], and has not assessed PA during PE using accelerometry [12].

The findings of this single school experiment indicated that primary school children only spend an average of 9.5% of their PE lessons engaged in MVPA, a figure much lower than the 30-40% of PE spent in MVPA in adolescents [10,15,16] and the recommended 50% threshold [10]. However, the only known study examining primary school PE found that 18% of lessons were spent in MVPA [18]. Whilst this figure is slightly higher than in the current study, both findings indicate that primary school PE is less effective at engaging children in MVPA than secondary school PE. In fact, evidence suggests that primary school PE achieves low standards in enabling young people to develop fitness [27]. Furthermore, in the study by Waring et al. [18] 46% of all PE lessons were spent developing motor skills and 18% watching and talking to teachers, despite almost two thirds of lessons consisting of team games. Whilst the percentage of time spent engaged in these types of activities were not measured in the current study, observation of the lessons highlighted that much time was spent listening to teachers and developing skills. Primary school PE lessons of this one class and one teacher seem to focus on skill development as the main aspect of PE [28]. Whilst this skill development is important for life-long PA participation [29], it may be restrictive of MVPA. However, opportunities for MVPA during PE are often underutilized. For example, instead of simply waiting in line to catch ball children should be provided with activities to perform PA whilst waiting (e.g. jogging on the spot). These activities will increase levels of PA alongside continued skill development.

The findings of this study also revealed that children were most active during team games PE lessons as opposed to movement PE lessons. Children spent 11.4% of team game lessons engaged in MVPA as opposed to 7.5% of movement lessons. These results concur with the findings of Fairclough and Stratton [10] and Waring et al. [18] who also found that more PA was performed during team games lessons than movement lessons. Team games require use of a significant proportion of muscle mass (e.g. running for the ball in football) perhaps therefore accounting for increased levels of MVPA [10]. As team games make up the majority of PE lessons, evidenced in the current study and that of Waring et al. [18]; it may also be that teachers are more skilled at delivering games activities [10]. However, despite the higher levels of MVPA during team games, levels of PA during PE in primary school children are still worryingly low. Interventions to increase PA during primary school PE lessons need to implemented and these should seek to target team games lessons as these make up the majority of the primary school PE curriculum and thus present the greatest potential for change. Whilst previous research has demonstrated that PE interventions can effectively increase PA [17], research is generally limited and has not focused on team games specifically. Teachers also need to be educated regarding best practice for teaching PE, or PE trained teachers employed, as it is possible that a lack of input within primary school teacher training limits teacher’s knowledge and understanding of what is required [18].

The secondary aim of this study was to compare PA levels during PE and school playtime. The comparison of PE lesson MVPA and playtime MVPA highlighted that children were significantly more active during PE, with only 6.1% of playtime being spent in MVPA. Whilst Waring et al. [18] reported a higher level of PA during playtime; the study also reported that children were more active during PE than playtime. In recent year’s school playtime has been highlighted as an important opportunity to allow children to engage in PA on a daily basis [11], yet the findings of this and other studies indicate that it is not effective at doing so and less effective than PE. Thus, steps need to be taken to provide children with opportunity to be more active. These steps could include strategies such as playground markings and equipment, set playtime activities and lunch time supervisors to provide children with active games to take part in [18,21]. Encouraging team games might be an effective strategy for playtime as team games are more facilitative of MVPA than movement activities and require increased use of muscle mass [10].

The current study has several limitations. Firstly, the use of only one class of children from one primary school may limit the application of the study to other primary schools and those within other regions of the UK. In addition the sample size was relatively small, particularly for analysis of playtime where there were only 12 participants. The limitations of using accelerometers also need to be considered. Accelerometers more accurately detect locomotive movements and less accurately detects upper body movements [30]. Thus if the movement lessons included more upper body activity than locomotion it is possible that MVPA was underestimated. The use of accelerometer cut points could also be considered as a limitation. There are a variety of cut points within the literature, all of which have been developed using a range of activities, criterion measures and age groups [31]. Whilst the most suitable cut points for the children and activities in the current study were selected, the variety of cut points limits the comparison of the output of one study to another.

## Conclusions

This study examined primary school children’s PA levels during different types of PE lessons and compared PE PA levels to those achieved during school playtime. The children in the current study only spent 9.5% of PE lessons engaged in MVPA and were significantly less active during movement lessons compared to team games. Furthermore, children were more active during PE than playtime. Both playtime and PE provide an important opportunity for PA however they are currently not being used effectively by primary school children. Interventions to target both areas of the school day are therefore required. Interventions during PE should target games lessons as they dominate the PE curriculum, encourage most PA and thus present the greatest potential for change. During playtime strategies to increase MVPA should encourage participation in team games through the provision of playground equipment, playground markings and set activities. It is essential that children’s PA during PE and playtime are enhanced to allow children to engage in appropriate amounts of PA for health. However, it might be more important to target school playtime, as this provides children with a daily opportunity for PA.

## Abbreviations

PA: Physical activity
MVPA: Moderate to vigorous physical activity
PE: Physical education
